# Perceived institutional support, physical activity engagement, and sociocultural adaptation among Chinese international students in Kazakhstan: a variable-and person-centered approach

**DOI:** 10.3389/fpsyg.2026.1854444

**Published:** 2026-09-25

**Authors:** Zheying Xiao, Kyrmyzy Zhumabekova, Rong Fan, Fan Jian, Lixue Huai, Shu Wen

**Affiliations:** 1 Al-Farabi Kazakh National University, Almaty, Kazakhstan; 2 School of International Communication and Language Intelligence, Wenhua College, Wuhan, China; 3 Kyrgyz International University, Bishkek, Kyrgyzstan; 4 Bishkek State University named after Academician Kusein Karasaev, Bishkek, Kyrgyzstan; 5 Kyrgyz National University named after Zhusup Balasagyn, Bishkek, Kyrgyzstan

**Keywords:** Chinese international students, Conservation of Resources theory, latent profile analysis, perceived institutional support, physical activity engagement, sociocultural adaptation, structural equation modeling

## Abstract

**Background:**

Sociocultural adaptation is a resource-demanding process for international students. Drawing on Conservation of Resources theory, this study examined perceived institutional support as a contextual resource and physical activity engagement as a behavioral resource-investment pathway among Chinese international students in Kazakhstan.

**Methods:**

A cross-sectional online survey was completed by 353 Chinese international students. Confirmatory factor analysis was used to examine the proposed measurement structure and construct distinction. A latent structural equation model tested the indirect association between perceived institutional support and sociocultural adaptation through physical activity engagement. Latent profile analysis identified student subgroups based on 15 perceived institutional support indicators, and the BCH procedure compared physical activity engagement and sociocultural adaptation across profiles.

**Results:**

The multidimensional CFA showed good fit, χ^2^(539) = 845.496, CFI = 0.982, TLI = 0.981, RMSEA = 0.040, and SRMR = 0.028, whereas the one-factor model showed poor fit. The SEM showed generally adequate fit across most indices, χ^2^(618) = 1012.610, CFI = 0.978, TLI = 0.976, RMSEA = 0.043, and SRMR = 0.094, although the SRMR was somewhat elevated. Perceived institutional support was positively associated with physical activity engagement (β = 0.494, *p* < 0.001), and physical activity engagement was positively associated with sociocultural adaptation (β = 0.198, *p* = 0.008). The standardized indirect effect was significant (β = 0.098, 95% CI [0.026, 0.198], *p* = 0.023), whereas the direct path from perceived institutional support to sociocultural adaptation was not significant after physical activity engagement was included. The latent profile analysis supported a four-profile solution: low support, academic-support-dominant, attachment-dominant, and high-support profiles. BCH tests showed significant profile differences in both physical activity engagement, χ^2^(3) = 31.443, *p* < 0.001, and sociocultural adaptation, χ^2^(3) = 18.215, *p* < 0.001.

**Conclusion:**

The findings are consistent with a resource-based indirect pathway linking perceived institutional support, physical activity engagement, and sociocultural adaptation. The person-centered results further show that students occupy unequal resource positions, suggesting that support services for international students should be differentiated rather than uniform.

## 1 Introduction

As international student mobility continues to expand, understanding how students adapt to unfamiliar academic, social, and cultural environments has become an important topic in educational and cross-cultural adjustment (Gaitán-Aguilar et al., 2022; Wang et al., 2018). Acculturation is commonly used as a broad term for the psychological and behavioral changes that occur through sustained intercultural contact (Berry, 1997, 2005). Within this broader process, sociocultural adaptation refers more specifically to the practical and behavioral competence required to manage everyday life in the host environment (Demes and Geeraert, 2015; Ward and Geeraert, 2016).

Universities are one of the most immediate environments in which international students encounter, lose, rebuild, and acquire resources. Academic guidance, campus-based social opportunities, accessible administrative services, and a sense of institutional belonging may all help students manage the uncertainty of study abroad (Xu and Chai, 2025). Previous research has linked supportive institutional and social environments to lower acculturative stress and more favorable academic and psychological adjustment among international students (Bai, 2016; Ng et al., 2017; Kristiana et al., 2022). However, studies have more often treated institutional support as a general predictor than as a multidimensional resource environment that may be experienced differently across students.

Physical activity engagement is another potentially relevant but still underexamined pathway in international student adaptation. In this study, the construct does not refer to objectively measured exercise frequency, duration, or intensity. Rather, it captures students’ commitment to and persistence in regular physical activity. Such engagement can create structured routines, increase opportunities for interaction, strengthen perceived competence, and contribute to psychological resources (Guerriero et al., 2025). For students adapting to a host environment, these resources may be particularly important because everyday sociocultural adjustment requires both external support and active behavioral participation (Gebregergis and Csukonyi, 2025; Khoirunnisa et al., 2025).

The present study is grounded in Conservation of Resources (COR) theory (Hobfoll, 1989, 2001, 2011). COR theory assumes that individuals strive to obtain, retain, protect, and build resources, especially when facing stress or environmental change. From this perspective, perceived institutional support can be viewed as a contextual resource, whereas physical activity engagement can be viewed as a behavioral resource-investment process. Sociocultural adaptation, in turn, reflects an adaptive outcome that may depend on both available resources and the capacity to mobilize them.

The theoretical contribution of the present study lies not only in testing associations among these constructs, but also in integrating variable-centered and person-centered approaches. The variable-centered SEM mediation model examines whether a general resource pathway links perceived institutional support to sociocultural adaptation through physical activity engagement. The person-centered latent profile model examines whether students occupy unequal institutional-resource positions. Integrating these two approaches makes it possible to examine both the process of resource transmission and the heterogeneity of resource distribution, thereby extending COR theory to the sociocultural adaptation of Chinese international students in a non-Western higher education context.

## 2 Theoretical foundations and hypotheses

### 2.1 Perceived institutional support and sociocultural adaptation

Sociocultural adaptation involves managing everyday interactions, communication, social norms, academic routines, and host-environment demands (Ward and Kennedy, 1999). For international students, this process can be accompanied by uncertainty, role ambiguity, and loss of familiar social and cultural resources when navigating new sociocultural environment (Bierwiaczonek and Waldzus, 2016; Chong et al., 2020; Schumann et al., 2020). COR theory suggests that external resources become particularly important under such conditions (Hobfoll, 1989, 2001). Perceived institutional support reflects students’ belief that their university provides useful academic, social, and institutional resources and values their needs as international students (Eisenberger et al., 1986). Universities provide academic guidance, social opportunities, and administrative assistance, all of which may help students cope with adaptation demands. Empirical studies have shown that support from institutional and social contexts is associated with reduced acculturative stress and improved adjustment outcomes (Bai, 2016; Kristiana et al., 2022; Chen et al., 2025). When students perceive these resources as available and accessible, they are more likely to engage with the host environment and adapt successfully. Based on this reasoning, the following hypothesis is proposed:

H1: Perceived institutional support is positively associated with sociocultural adaptation.

### 2.2 Perceived institutional support and physical activity engagement

Institutional support may also be related to students’ active engagement in health-related and campus-based behaviors. A supportive university environment can provide access to facilities, social encouragement, organized activities, and a sense of safety and belonging (Chaudhry et al., 2024). Physical activity engagement, defined here as commitment to and persistence in regular activity, is therefore expected to be higher when students perceive stronger institutional support (Han et al., 2025).

Within COR theory, these contextual resources may enable students to invest energy in activities that generate further resources. Previous research has consistently shown that supportive contexts, including social and environmental resources, are associated with higher levels of physical activity (Laird et al., 2018; Lee et al., 2020; Niu et al., 2023). More recent studies further report that perceived institutional support acts as a critical resource that fosters motivation, self-efficacy, and sustained behavioral engagement in physical activity across different age groups and educational settings (Guo et al., 2023; Lin et al., 2025; Huang et al., 2025; Meerits et al., 2025; Xiong et al., 2025).Accordingly, the following hypothesis is proposed:

H2: Perceived institutional support is positively associated with physical activity engagement.

### 2.3 Physical activity engagement and sociocultural adaptation

Physical activity engagement is theorized to support sociocultural adaptation because it functions as a proactive investment of such resources rather than solely a health behavior. This reasoning follows Conservation of Resources theory (Hobfoll, 1989, 2011), which holds that individuals invest available resources in activities that may generate further gains. Engaging in physical activity may generate multiple forms of resources. It can improve psychological well-being, enhance perceived competence, and create opportunities for social interaction. These outcomes are particularly relevant in sociocultural contexts, where individuals must establish new social connections and adapt to unfamiliar norms. Empirical research has shown that physical activity engagement is associated with improved well-being, reduced stress, and better adjustment among diverse populations (Gerber et al., 2012; Camplain et al., 2020; Zhu et al., 2021; Zan and Fan, 2018). Studies focusing on immigrant groups further suggest that this engagement is also linked to sociocultural processes, including social integration and environmental engagement (Pichon et al., 2007; Dellaserra et al., 2018; Congello et al., 2019). More recent empirical work shows that physical activity provides opportunities for interaction that do not depend on language fluency or prior cultural familiarity, and has been shown to strengthen broader social adjustment through peer attachment (Xing and Ge, 2025; Zhou et al., 2024). Among international students specifically, qualitative longitudinal evidence indicates that shared physical activity was a primary avenue through which participants formed cross-cultural friendships and eased social integration during their first year abroad (Li and Zizzi, 2017).

Evidence linking physical or sport activity directly to international students’ adaptation outcomes remains limited but broadly supportive. Accordingly, physical activity engagement is hypothesized to be positively associated with sociocultural adaptation among international students. Thus, the following hypothesis is proposed:

H3: Physical activity engagement is positively associated with sociocultural adaptation.

### 2.4 The mediating role of physical activity engagement

Building on the preceding arguments, physical activity engagement may also function as an intermediary mechanism linking perceived institutional support to sociocultural adaptation. Within COR theory, this reflects a resource transmission process, in which initial resources enable behaviors that generate additional resources, ultimately leading to improved outcomes. Specifically, perceived institutional support provides individuals with access to contextual resources that facilitate engagement in physical activity. Through participation in physical activity, individuals may accumulate psychosocial resources, such as social connections and psychological resilience, which in turn support sociocultural adaptation. This sequence is consistent with the concept of resource gain spirals, whereby initial resource availability leads to further resource acquisition (Hobfoll, 2011).

Empirical evidence supports the plausibility of this pathway. Studies have shown that support systems influence adaptation through behavioral and environmental mechanisms (Ward and Geeraert, 2016; Schumann et al., 2020). In addition, research has demonstrated that physical activity engagement is linked to both contextual factors and adaptation outcomes (Camplain et al., 2020; Zhu et al., 2021).

Accordingly, the following hypothesis is proposed:

H4: Physical activity engagement mediates the relationship between perceived institutional support and sociocultural adaptation.

### 2.5 Latent profiles of perceived institutional support

Variable-centered models estimate average associations across the sample, but they do not show whether students experience similar or different configurations of institutional resources. Person-centered approaches such as latent profile analysis are useful for identifying subgroups that share similar response patterns (Howard and Hoffman, 2018). From a COR perspective, such profiles can represent unequal resource positions. Some students may report broad support across academic, social, and attachment domains, whereas others may report more uneven configurations.

H5: Distinct latent profiles of perceived institutional support can be identified among Chinese international students.

### 2.6 Differences across latent profiles

COR theory suggests that individuals with greater resource availability are better equipped to cope with stress and achieve positive outcomes (Hobfoll, 2001). If latent profiles represent different institutional-resource positions, they should also differ in outcomes related to resource investment and adaptation. Students in profiles with stronger or broader support may show higher physical activity engagement and more favorable sociocultural adaptation. Students with limited support may be at greater risk of lower engagement and poorer adaptation. Empirical research has shown that differences in resource contexts are associated with variation in acculturation outcomes (Ng et al., 2017; Kristiana et al., 2022).

H6: Latent profiles of perceived institutional support differ significantly in physical activity engagement and sociocultural adaptation.

## 3 Materials and methods

### 3.1 Participants and procedure

Participants were Chinese international students enrolled at universities in Kazakhstan. Data were collected through an online survey distributed via university-related networks and Chinese student groups. Because recruitment relied on network-based distribution, the number of students who received the survey invitation could not be determined precisely. Participation was voluntary and anonymous. Before completing the survey, students were informed of the study purpose and provided informed consent. A total of 382 responses were initially obtained. Data were screened before analysis for consent, completeness, duplicate or highly similar records where metadata allowed, implausible demographic entries, unrealistically short completion patterns, and invariant or patterned responses. After excluding incomplete or invalid responses, 353 valid cases were retained. The analytic dataset contained no missing values on the variables used in the CFA, SEM, LPA, and BCH analyses.

The final sample had a mean age of 23.38 years. Among the participants, 193 were male (54.7%) and 160 were female (45.3%). In terms of education level, 137 were undergraduate students (38.8%), 121 were master’s students (34.3%), and 95 were doctoral students (26.9%). Regarding length of stay in Kazakhstan, 73 students (20.7%) had stayed for 1 year or less, 168 (47.6%) for 1 to 2 years, and 112 (31.7%) for more than 2 years. Local language proficiency was reported as low by 65 students (18.4%), moderate by 185 students (52.4%), and high by 103 students (29.2%).

### 3.2 Measures

#### 3.2.1 Perceived institutional support

Perceived institutional support was assessed using 15 items adapted from the institutional and environmental components of the Student Adaptation to College Questionnaire (Baker and Siryk, 1984; Credé and Niehorster, 2012). Because the SACQ was designed to assess student adjustment rather than perceived institutional support, the adaptation involved content screening and reconceptualization: only content referring to university-provided academic resources, opportunities for social connection, or institutional connection was retained, and wording was modified so that the university or university community was the referent. Accordingly, this measure represents a study-specific operationalization of perceived institutional support rather than direct administration of an established perceived institutional support scale. In this study, perceived institutional support was defined as students’ subjective evaluation of the academic, social, and institutional resources provided by their university. The measure included three domains: academic support (5 items), social support (5 items), and institutional attachment (5 items). Example items included “I can easily access the learning resources necessary for my studies,” “I feel that I am part of the university community,” and “I am proud to be a student at this university.” Items were rated on a five-point scale from 1 (strongly disagree) to 5 (strongly agree), with higher scores indicating stronger perceived institutional support.

For transparency and replication, the complete 15-item wording used in the survey was as follows. Academic support: (1) “My university provides well-organized courses that support my learning needs”; (2) “Faculty members are willing to provide academic guidance when I need it”; (3) “I can easily access the learning resources necessary for my studies”; (4) “My university offers sufficient academic support services”; and (5) “Overall, I am satisfied with the academic support provided by my university.” Social support: (6) “My university provides opportunities for students to interact and build social connections”; (7) “My university helps international students adapt to campus life”; (8) “I feel that I am part of the university community”; (9) “University-organized activities and events help me adjust socially”; and (10) “I can receive social support on campus when needed.” Institutional attachment: (11) “I feel a sense of belonging to my university”; (12) “I am proud to be a student at this university”; (13) “If I had to choose again, I would still choose to study at this university”; (14) “My university cares about students’ well-being and development”; and (15) “Overall, I am satisfied with the support provided by my university.”

#### 3.2.2 Physical activity engagement

Physical activity engagement was assessed with eight items adapted from the Physical Activity Scale developed by Wu et al. (2016). The measure included two domains: commitment to physical activity (4 items) and persistence in physical activity (4 items). Because the scale assesses commitment and persistence rather than objectively measured frequency, duration, or intensity, the construct is referred to as physical activity engagement. A sample item is “I make time for physical activity even when I am busy.” Items were rated on a five-point scale from 1 (strongly disagree) to 5 (strongly agree), with higher scores indicating stronger engagement in physical activity.

#### 3.2.3 Sociocultural adaptation

Sociocultural adaptation was assessed using the Brief Sociocultural Adaptation Scale (BSAS; Demes and Geeraert, 2015). The BSAS is a measure to assess individuals’ sociocultural adaptation in a host-country setting. The scale assesses perceived ease or difficulty in managing everyday social and cultural demands in the host environment. The BSAS consists of 12 items. Participants were asked to reflect on their experiences in the new sociocultural environment and report how easy or difficult it was for them to adapt to the local environment. Sample items included adaptation to the “Climate (temperature, rainfall, and humidity),” “Speaking and communicating in the local environment” and “Customs and everyday social norms.” Items were rated on a seven-point scale from 1 (very difficult) to 7 (very easy), with higher scores indicating better sociocultural adaptation. The scale was administered in Chinese after translation and back-translation, with minor wording adjustments to improve contextual clarity for Chinese international students in Kazakhstan.

#### 3.2.4 Control variables

Based on prior research and the preliminary group-difference analyses, length of stay in Kazakhstan and local language proficiency were included as control variables in the SEM mediation model. Gender and education level were described but were not retained as controls because they were not significantly associated with the main variables in preliminary analyses.

### 3.3 Data analysis

Data were analyzed using SPSS and Mplus. Descriptive statistics, skewness, kurtosis, and bivariate correlations were first calculated for perceived institutional support, physical activity engagement, and sociocultural adaptation. Independent-samples *t* tests and one-way ANOVAs were used to examine group differences across demographic variables.

To evaluate the proposed measurement structure and construct distinction, CFA models were estimated in Mplus. First, a one-factor model in which all items loaded on a single common factor was tested. Second, a multidimensional CFA was estimated, specifying perceived institutional support as academic support, social support, and institutional attachment; physical activity engagement as commitment and persistence; and sociocultural adaptation as two first-order domains. Model fit was evaluated using χ^2^, CFI, TLI, RMSEA, and SRMR. Composite reliability and average variance extracted were calculated from standardized factor loadings. Evidence relevant to discriminant validity in the present sample was evaluated through model comparison and the pattern of latent factor correlations.

A SEM mediation model was then estimated using the multidimensional measurement structure supported by the CFA. Perceived institutional support, physical activity engagement, and sociocultural adaptation were modeled as higher-order latent constructs. The indirect effect was tested using 5,000 bootstrap samples. Because the data are cross-sectional, the mediation model was interpreted as evidence of an indirect association rather than a causal pathway.

Latent profile analysis was conducted using the 15 perceived institutional support items. Models with one to six profiles were compared using AIC, BIC, sample-size adjusted BIC, entropy, LMRT, BLRT, class sizes, average posterior probabilities, parsimony, and theoretical interpretability. The BCH method was then used to compare physical activity engagement and sociocultural adaptation across the retained profiles while accounting for classification uncertainty (Asparouhov and Muthén, 2014).

### 3.4 Common method bias

Because all variables were collected through self-report, common method bias was considered. Harman’s single-factor approach was not treated as definitive because this test has known limitations and cannot by itself rule out method effects. Therefore, we compared the one-factor CFA with the multidimensional CFA. The one-factor model showed very poor fit, whereas the multidimensional model showed good fit, suggesting that the main constructs were empirically distinguishable and that a single common factor did not adequately account for the item covariance structure. Nevertheless, the use of self-report data remains a limitation, and the results should be interpreted with this constraint in mind.

## 4 Results

### 4.1 Descriptive statistics and correlations

Table 1 presents descriptive statistics and correlations among the main variables. Perceived institutional support was positively correlated with physical activity engagement (*r* = 0.315, *p* < 0.01) and sociocultural adaptation (*r* = 0.253, *p* < 0.01). Physical activity engagement was also positively linked to sociocultural adaptation (*r* = 0.251, *p* < 0.01). Skewness and kurtosis values suggested no severe deviation from normality for the composite variables.

**TABLE 1:** Descriptive statistics and correlations among the main variables.

Variable	M	SD	Skewness	Kurtosis	1	2	3
1. Perceived institutional support	3.09	0.92	−0.174	−1.096	−	−	–
2. Physical activity engagement	2.54	1.24	0.398	−0.993	0.315**		
3. Sociocultural adaptation	4.34	1.63	−0.563	−0.748	0.253**	0.251**	

*N =* 353. **p < 0.01. Scores for perceived institutional support and physical activity engagement ranged from 1 to 5; sociocultural adaptation ranged from 1 to 7.

### 4.2 Demographic group differences

Preliminary group comparisons are shown in Table 2. Gender and education level were not significantly associated with perceived institutional support, physical activity engagement, or sociocultural adaptation. Length of stay in Kazakhstan and local language proficiency showed significant differences across all three variables and were therefore retained as controls in the SEM mediation model.

**TABLE 2:** Demographic characteristics and group differences.

Variable	*n*	%	PIS t/F	PAE t/F	SA t/F
Gender			0.919	1.148	−0.766
Male	193	54.7			
Female	160	45.3			
Education level			0.093	0.165	0.021
Bachelor	137	38.8			
Master	121	34.3			
Doctoral	95	26.9			
Length of stay			7.582**	4.893**	190.794***
≤ 1 year	73	20.7			
1–2 years	168	47.6			
≥ 2 years	112	31.7			
Local language proficiency			7.146**	5.796**	186.196***
Low	65	18.4			
Moderate	185	52.4			
High	103	29.2			

PIS, perceived institutional support; PAE, physical activity engagement; SA, sociocultural adaptation. **p < 0.01; ***p < 0.001.

### 4.3 Measurement structure and construct distinction

The one-factor CFA showed very poor fit, χ^2^(560) = 12407.542, CFI = 0.320, TLI = 0.277, RMSEA = 0.245, and SRMR = 0.234. By contrast, the multidimensional CFA showed good fit, χ^2^(539) = 845.496, CFI = 0.982, TLI = 0.981, RMSEA = 0.040, and SRMR = 0.028. This comparison supports the empirical distinction among perceived institutional support, physical activity engagement, and sociocultural adaptation (see Table 3).

**TABLE 3:** CFA model comparison.

Model	χ^2^	df	CFI	TLI	RMSEA	SRMR	AIC	BIC
One-factor model	12407.542	560	0.320	0.277	0.245	0.234	40296.662	40702.641
Multidimensional CFA	845.496	539	0.982	0.981	0.040	0.028	28776.616	29263.791

The one-factor model loaded all items onto a single factor. The CFA specified multidimensional measurement structures for main variables.

As shown in Table 4, standardized factor loadings ranged from 0.829 to 0.970 and were all statistically significant. Composite reliability values ranged from 0.944 to 0.988, and AVE values ranged from 0.772 to 0.930, providing evidence of convergent validity in the present sample. The latent factor correlations in the CFA ranged from 0.006 to 0.604, remaining below common thresholds for problematic construct overlap. Together with the poor one-factor model fit, these results provide evidence of discriminant validity in the present sample and support the empirical distinction among perceived institutional support, physical activity engagement, and sociocultural adaptation. The measurement results also support the interpretation of physical activity as engagement, with distinct commitment and persistence dimensions.

**TABLE 4:** Reliability, standardized loadings, CR, and AVE.

Latent factor	Items	α	Standardized loading range	CR	AVE
Academic support	PIS1–PIS5	0.958	0.895–0.916	0.958	0.819
Social support	PIS6–PIS10	0.945	0.829–0.912	0.946	0.777
Institutional attachment	PIS11–PIS15	0.944	0.841–0.890	0.944	0.772
PA commitment	PAE1–PAE4	0.957	0.898–0.933	0.957	0.848
PA persistence	PAE5–PAE8	0.967	0.924–0.966	0.968	0.882
Sociocultural adaptation A	SA1–SA6	0.988	0.958–0.970	0.988	0.930
Sociocultural adaptation B	SA7–SA12	0.987	0.949–0.970	0.987	0.927

CR, composite reliability; AVE, average variance extracted. All standardized loadings were significant at *p* < 0.001.

### 4.4 SEM mediation analysis

The SEM mediation model was estimated using the multidimensional measurement structure identified in the CFA. The model showed generally adequate fit across most indices, χ^2^(618) = 1012.610, CFI = 0.978, TLI = 0.976, RMSEA = 0.043, 90% CI [0.038, 0.047], and SRMR = 0.094. The SRMR value was slightly above the conventional 0.080 threshold, suggesting that some residual covariance may remain unmodeled; however, the CFI, TLI, and RMSEA values supported the adequacy of the model for testing the proposed indirect association. The SEM mediation results are presented in Table 5.

**TABLE 5:** SEM mediation results.

Path/effect	β	SE	*p*	95% CI
PIS → PAE	0.494	0.076	< 0.001	–
PAE → SA	0.198	0.075	0.008	–
PIS → SA direct path	0.057	0.071	0.416	–
Stay length → SA	0.472	0.079	< 0.001	–
Language proficiency → SA	0.389	0.082	< 0.001	–
PIS → PAE → SA indirect effect	0.098	0.043	0.023	[0.026, 0.198]
Total effect PIS → SA	0.155	0.057	0.007	[0.044, 0.267]

Bootstrap confidence intervals were based on 5,000 samples. PIS, perceived institutional support; PAE, physical activity engagement; SA, sociocultural adaptation.

The standardized path coefficients are presented in Figure 1. Standardized path coefficients indicated that perceived institutional support was positively associated with physical activity engagement, β = 0.494, *p* < 0.001. Physical activity engagement was positively related with sociocultural adaptation, β = 0.198, *p* = 0.008. After physical activity engagement was included, the direct association between perceived institutional support and sociocultural adaptation was not significant, β = 0.057, *p* = 0.416. The standardized indirect effect was significant, β = 0.098, 95% CI [0.026, 0.198], *p* = 0.023. These findings support the proposed indirect association, while the cross-sectional design precludes causal interpretation.

**FIGURE 1:**
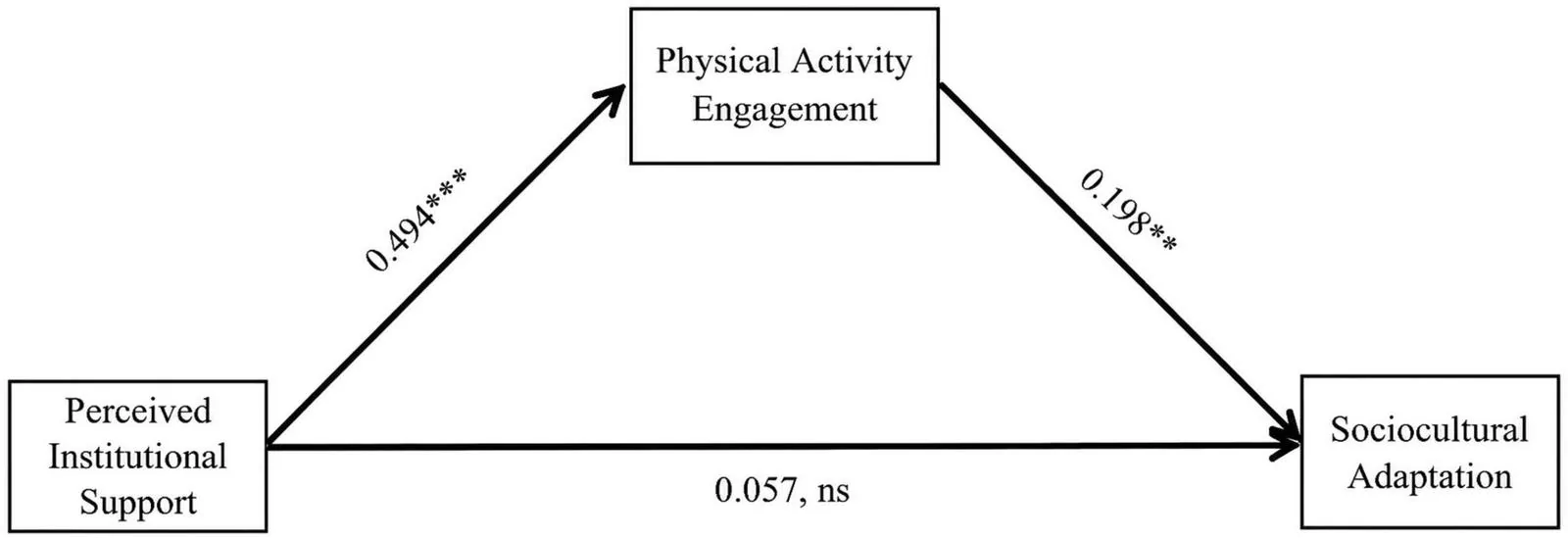
Revised SEM mediation model. PIS, perceived institutional support; PAE, physical activity engagement; SA, sociocultural adaptation. Standardized coefficients are shown. Stay length and local language proficiency were controlled but omitted from the diagram for visual clarity. **p < 0.01; ***p < 0.001; ns, not significant.

### 4.5 Latent profile analysis

LPA models were estimated using the 15 perceived institutional support items. Fit indices for the one- to five-profile models are presented in Table 6. Although AIC, BIC, and adjusted BIC continued to decrease with additional profiles, the LMRT was significant through the four-profile solution and became non-significant for the five- profile solution. The four-profile solution also showed excellent entropy (0.992), acceptable class sizes, strong average posterior probabilities, and the clearest theoretical interpretability. The five- profile solutions mainly split existing profiles into narrower subgroups without adding a substantively clearer resource configuration. Therefore, the four-profile solution was retained as the most parsimonious and interpretable model.

**TABLE 6:** Latent profile model fit indices.

Profiles	AIC	BIC	aBIC	Entropy	LMRT	BLRT	Class proportions (%)
1	17866.967	17982.962	17887.789	–	–	–	100.0
2	15421.487	15599.345	15453.414	0.976	< 0.001	< 0.001	48.2/51.8
3	14839.372	15079.093	14882.403	0.986	0.0003	< 0.001	13.9/35.1/51.0
4	13999.822	14301.407	14053.959	0.992	< 0.001	<0.001	30.0/12.7/17.6/39.7
5	13602.610	13966.058	13667.852	0.989	0.2192	< 0.001	12.2/19.3/11.6/39.9/17.0

aBIC, sample-size adjusted BIC; LMRT, Lo-Mendell-Rubin adjusted likelihood ratio test; BLRT, bootstrap likelihood ratio test.

The four retained profiles were labeled according to their response patterns. Class 1, the low-support profile, represented 106 students (30.0%) and showed low academic support, moderate social support, and low institutional attachment. Class 2, the academic-support-dominant profile, represented 45 students (12.7%) and showed high academic support but low institutional attachment. Class 3, the attachment-dominant profile, represented 62 students (17.6%) and showed low academic support but relatively high institutional attachment. Class 4, the high-support profile, represented 140 students (39.7%) and showed high scores across academic support, social support, and institutional attachment. Average posterior probabilities were excellent: 0.997 for C1, 1.000 for C2, 0.999 for C3, and 0.994 for C4. The response patterns of the four retained profiles are illustrated in Figure 2.

**FIGURE 2:**
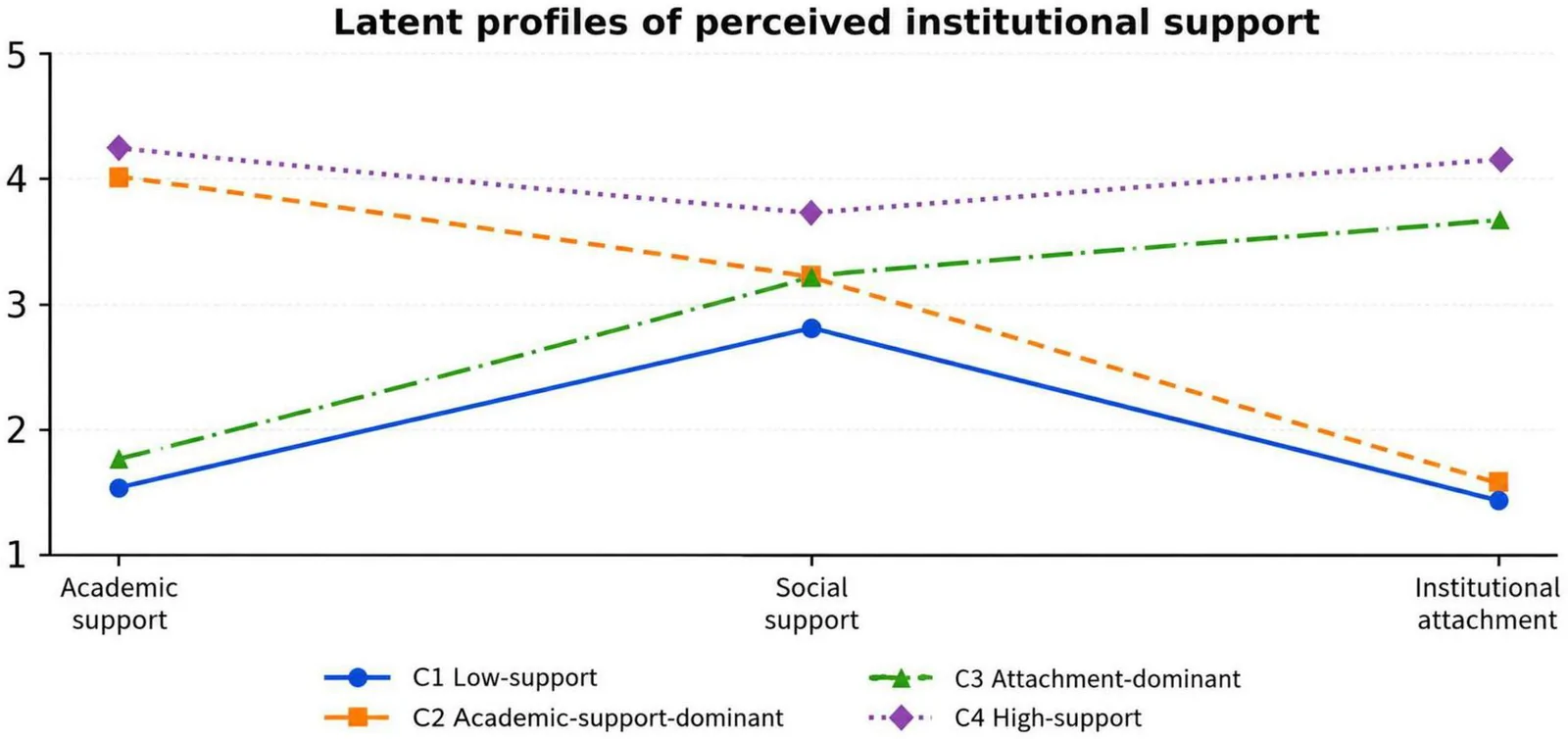
Latent profiles of perceived institutional support.

### 4.6 BCH profile comparisons

The BCH method was used to compare physical activity engagement and sociocultural adaptation across the four profiles. Significant overall profile differences were found for physical activity engagement, χ^2^(3) = 31.443, *p* < 0.001. The high-support profile reported the highest physical activity engagement, followed by the academic-support-dominant profile, the attachment-dominant profile, and the low-support profile. Pairwise comparisons showed that the high-support profile had significantly higher physical activity engagement than the low-support and attachment-dominant profiles. The academic-support-dominant profile also showed higher physical activity engagement than the low-support profile. The BCH profile comparison results are presented in Table 7.

**TABLE 7:** Group differences across the four profiles.

Variable	C1 low-support	C2 academic-support-dominant	C3 attachment-dominant	C4 high-support	χ^2^(3)	*p*
Physical activity engagement	2.169 ± 0.093	2.575 ± 0.179	2.224 ± 0.138	2.956 ± 0.115	31.443	< 0.001
Sociocultural adaptation	3.836 ± 0.168	4.369 ± 0.269	4.329 ± 0.182	4.725 ± 0.125	18.215	< 0.001

Values are BCH-estimated means ± standard errors. Pairwise tests showed that for PAE, C4 > C1, C4 > C3, and C2 > C1. For SA, C4 > C1 and C3 > C1.

Significant overall profile differences were also found for sociocultural adaptation, χ^2^(3) = 18.215, *p* < 0.001. The high-support profile reported the highest sociocultural adaptation score, whereas the low-support profile reported the lowest. Pairwise comparisons indicated that the high-support and attachment-dominant profiles had significantly higher sociocultural adaptation than the low-support profile. Not all pairwise comparisons were significant, suggesting that profile differences were mainly driven by the contrast between students with limited institutional resources and those with stronger institutional attachment or broader support.

## 5 Discussion

### 5.1 Main findings and theoretical contribution

This study examined how perceived institutional support, physical activity engagement, and sociocultural adaptation are related among Chinese international students in Kazakhstan. The analyses suggest that the relationship is not best understood as a simple direct-support model. Instead, perceived institutional support was associated with sociocultural adaptation through a significant indirect pathway involving physical activity engagement. The standardized indirect effect was modest but significant, β = 0.098, 95% CI [0.026, 0.198], *p* = 0.023, whereas the direct path from perceived institutional support to sociocultural adaptation was not significant after physical activity engagement was included. This pattern is best described as an indirect-only effect in the present SEM model rather than causal full mediation, because the data were cross-sectional.

This finding refines, rather than replaces, prior work on support and international student adaptation. Previous studies have often treated social or institutional support as a protective condition in international students’ acculturative stress, university adjustment, and adaptation processes (Bai, 2016; Kristiana et al., 2022; Chen, 2025). The present study complicates this view by suggesting that perceived support may be more strongly connected with adaptation when it is mobilized through students’ engagement in structured, resource-generating activities. This does not mean that institutional support lacks direct relevance. Rather, it indicates that, in this sample and model, the measurable link between institutional support and sociocultural adaptation was largely carried through physical activity engagement.

The interpretation is consistent with Conservation of Resources theory, but the mechanism requires precision. In this study, physical activity engagement is conceptualized as a form of resource investment because it requires students to invest time, energy, self-regulation, and social exposure in a structured activity context. Such investment may generate additional resources, including routine, perceived competence, emotional regulation, peer contact, and familiarity with the host environment (Hobfoll, 1989, 2001, 2011). Thus, institutional support is treated as a contextual resource, physical activity engagement as an investment process, and sociocultural adaptation as an adaptation-related outcome. This mechanism was hypothesized from COR theory, whereas the more fine-grained profile interpretation from the LPA should be viewed as an exploratory person-centered extension of the same theoretical framework.

The Kazakhstan context further strengthens the relevance of this resource-based interpretation. Research on international students in Kazakhstan indicates that adaptation involves educational, sociocultural, language-related, and interpersonal challenges (Kaikenov et al., 2024). Kazakhstan higher education also operates within a multilingual environment involving Kazakh, Russian, and increasingly English, which may intensify everyday communication and academic-literacy demands for foreign students (Yermekova et al., 2024). For Chinese students in this context, institutional support may become adaptive not only by being available, but by helping students enter structured spaces where language practice, peer contact, routine, and participation can occur. Physical activity engagement may represent one such space.

The second contribution of the study is methodological and theoretical. By integrating SEM and LPA, the study shows both a general indirect pathway and heterogeneous institutional-support configurations. The LPA identified four profiles: low-support, academically supported, institutionally attached, and high-support profiles. These profiles show that students are not equally positioned within the resource pathway. Some students reported broad institutional support, whereas others reported uneven configurations, such as strong academic support but weak institutional attachment, or strong attachment but weak academic support. This person-centered evidence extends COR theory by linking resource transmission with unequal and unevenly distributed resource configurations (Hobfoll, 2001, 2011; Howard and Hoffman, 2018).

### 5.2 Variable-centered pathway: from institutional support to adaptation through engagement

The SEM results support the core variable-centered pathway proposed in the study. Perceived institutional support was positively associated with physical activity engagement, β = 0.494. Using conventional behavioral-science benchmarks for standardized effects, this path is close to a large association, although such benchmarks should be interpreted cautiously because effect sizes depend on measurement and context (Cohen, 1988). Substantively, the finding suggests that students who perceive stronger academic support, social support, and institutional attachment are more likely to report commitment to and persistence in physical activity.

This result is consistent with studies showing that supportive environments and social resources are associated with stronger physical activity participation or engagement (Laird et al., 2018; Lee et al., 2020; Niu et al., 2023). However, it is important to distinguish engagement from objective physical activity behavior. The measurement model indicated that the physical activity construct in this study reflected commitment and persistence rather than frequency, duration, intensity, or device-assessed activity. Therefore, the finding should be interpreted as evidence that perceived institutional support is associated with stronger physical activity engagement tendencies, not necessarily with greater objectively measured activity volume.

Physical activity engagement was also positively associated with sociocultural adaptation, β = 0.198. This association was smaller than the path from institutional support to physical activity engagement but remained statistically significant. The magnitude suggests that physical activity engagement is a meaningful but not dominant explanatory pathway. This interpretation is consistent with prior research linking physical activity with acculturation-related or adaptation-related outcomes among migrant and international populations (Gerber et al., 2012; Camplain et al., 2020; Zhu et al., 2021). At the same time, previous evidence also suggests that these associations can vary by population, host context, and measurement approach, which means that physical activity should not be treated as a universal adaptation mechanism. For example, research using both self-reported and accelerometer-assessed indicators has shown that acculturation-related associations may differ across activity domains and measurement methods, indicating that physical activity should not be treated as a uniformly beneficial or universal adaptation pathway (Camplain et al., 2020).

The indirect effect from perceived institutional support to sociocultural adaptation through physical activity engagement was statistically significant but modest. This is important for interpretation. The result supports an indirect-only pattern in the present SEM model, but it does not show that physical activity engagement is the only mechanism connecting institutional support and adaptation. Other pathways may operate alongside it, including campus belonging, academic self-efficacy, peer support, language confidence, psychological resilience, and acculturative stress. Moreover, because the study was cross-sectional, the temporal sequence cannot be established. Students who are already better adapted may also be more willing to engage in physical activity and may evaluate their institutions more positively. Longitudinal research is therefore needed before stronger mediation claims can be made.

### 5.3 Person-centered profiles and implications for institutional practice

Consistent with the model-comparison results reported above, the LPA identified four profiles of perceived institutional support. The high-support profile was the largest group, accounting for 39.7% of the sample, followed by the low-support profile at 30.0%, the attachment-dominant profile at 17.6%, and the academic-support-dominant profile at 12.7%. Classification quality was high, with entropy = 0.992, indicating that the profiles were well separated. These class sizes suggest that the profiles were substantively interpretable.

The profile structure shows that institutional support is not a uniform experience. The low-support profile was characterized by generally weak perceived support, particularly low academic support and low institutional attachment. The academic-support-dominant profile showed high academic support but low institutional attachment, suggesting that some students may view their university as academically useful without feeling emotionally connected to it. The attachment-dominant profile showed the opposite configuration: stronger institutional attachment but weaker academic support. The high-support profile showed consistently strong support across academic, social, and attachment dimensions.

The BCH results showed significant overall profile differences in both physical activity engagement and sociocultural adaptation. The high-support profile scored about 0.79 points higher than the low-support profile in physical activity engagement on a 1–5 scale and about 0.89 points higher in sociocultural adaptation on a 1–7 scale. These raw-scale differences suggest practical as well as statistical relevance. However, the pairwise results were not uniformly significant. For physical activity engagement, the high-support profile was significantly higher than the low-support and attachment-dominant profiles, and the academic-support-dominant profile was higher than the low-support profile. For sociocultural adaptation, the high-support and attachment-dominant profiles were significantly higher than the low-support profile, whereas several other contrasts were not significant. Thus, the clearest and most defensible interpretation is that students with broad or attachment-related institutional resources differed from those with limited perceived support, rather than that all four profiles formed a strict rank order.

This distinction matters for practice. The profiles should be used as a screening framework for identifying likely support gaps rather than as four rigid intervention categories. Students in the low-support profile may need broad resource building, including academic guidance, institutional outreach, peer mentoring, and structured opportunities for participation. For the academic-support-dominant profile, belonging-oriented programming may be more appropriate because academic support was relatively strong but institutional attachment was weak. For the attachment-dominant profile, academic-resource navigation may be more relevant because attachment was present but perceived academic support was limited. For the high-support profile, institutions may focus on maintaining resource access and involving these students in peer-support or engagement activities.

Physical activity programs may be useful because they can connect several resource domains at once. Campus sport, recreational exercise, walking groups, and informal activity programs can provide routine, low-stakes peer contact, and participation in the host environment. For international students, such programs may function not only as health-promotion activities but also as accessible spaces for social integration and sociocultural learning (Gerber et al., 2012; Elshahat et al., 2023). This implication follows directly from both sets of findings: physical activity engagement formed part of the indirect pathway, and the high-support profile showed the highest level of physical activity engagement.

### 5.4 Strengths, limitations, and future directions

This study has several strengths. First, it focuses on Chinese international students in Kazakhstan, a context that remains underrepresented in research on international student adaptation. This expands the literature beyond the more commonly studied Western host countries and adds evidence from a Central Asian higher education setting. Second, the study integrates SEM and LPA. The SEM model identifies a general indirect pathway, whereas the LPA model reveals heterogeneous institutional-support configurations. Third, the analyses strengthen the methodological rigor of the manuscript by adding CFA model comparison, factor loadings, latent factor correlations, CR, AVE, SEM mediation, corrected LPA, and BCH distal outcome tests.

Several limitations should be acknowledged. First, the cross-sectional design prevents causal inference. Although the hypothesized pathway is grounded in COR theory, alternative directions remain possible. Students with better sociocultural adaptation may become more physically active and may evaluate their institutions more positively. Future studies should use longitudinal, cross-lagged, or intensive longitudinal designs to examine temporal ordering. Second, all variables were measured through self-report, which may introduce common method bias and response bias. The poor one-factor CFA result and the strong fit of the multidimensional measurement model suggest that the constructs were empirically distinguishable. This point should be interpreted cautiously because single-factor approaches are limited as tests of common method bias (Podsakoff et al., 2003). Future research should use stronger procedural and statistical controls, such as temporal separation, multi-source data, marker variables, or latent method-factor approaches. Third, physical activity engagement was measured as commitment and persistence rather than objective behavior. Future studies should combine engagement scales with frequency-duration-intensity measures, activity logs, accelerometers, or wearable-device data. This would help distinguish students’ willingness to sustain physical activity from their actual activity volume. Fourth, perceived institutional support was adapted from SACQ-related content. Although the CFA provided evidence supporting the three-dimensional structure in this sample, the measure should be further validated in independent samples and other host-country contexts. Future studies should also test measurement invariance across gender, degree level, length of stay, and academic discipline, because these factors may shape both perceived support and adaptation experiences. Fifth, the sample size was acceptable for the present analyses, but person-centered models are sensitive to subgroup size and sample composition. The smallest profile accounted for 12.7% of the sample, which was interpretable but relatively small. Replication with larger samples would increase confidence in the stability of the four-profile solution. Although gender, education level, length of stay, and local language proficiency were described and statistically controlled where appropriate, the sample size did not allow robust profile-specific subgroup analyses across these demographic categories. Finally, recruitment relied on network-based online distribution, so the sampling frame and response rate could not be precisely estimated. The findings should therefore be generalized cautiously beyond Chinese international students in Kazakhstan. Future research should examine whether the same indirect pathway and support profiles appear among other international student groups, host countries, and institutional systems.

## 6 Conclusion

This study provides an account of the relationship among perceived institutional support, physical activity engagement, and sociocultural adaptation among Chinese international students in Kazakhstan. The SEM results are consistent with an indirect resource-based pathway in which perceived institutional support is associated with stronger physical activity engagement, which in turn is associated with better sociocultural adaptation. The LPA further shows that students occupy different institutional-resource positions, and these profiles differ in physical activity engagement and sociocultural adaptation. These findings suggest that universities should provide differentiated support strategies that strengthen academic support, social connection, institutional belonging, and behavioral engagement opportunities for international students.

## Data Availability

The raw data supporting the conclusions of this article will be made available by the authors, without undue reservation.
